# An individual-supported program to enhance placement in a sheltered work environment of autistic individuals mostly with intellectual disability: a prospective observational case series in an Italian community service

**DOI:** 10.3389/fpsyt.2023.1225236

**Published:** 2023-11-02

**Authors:** Roberta Maggio, Laura Turriziani, Caterina Campestre, Marcella Di Cara, Emanuela Tripodi, Caterina Impallomeni, Angelo Quartarone, Claudio Passantino, Francesca Cucinotta

**Affiliations:** ^1^Center for Autism “Dopo di noi”, Barcellona Pozzo di Gotto, Messina, Italy; ^2^Unit of Child Neurology and Psychiatry, Department of Human Pathology of the Adult and Developmental Age, “Gaetano Barresi” University of Messina, Messina, Italy; ^3^IRCCS Centro Neurolesi “Bonino-Pulejo”, Messina, Italy

**Keywords:** autism spectrum disorder, intellectual disability, employment, vocational/labor participation, transitional age, adolescents, adult

## Abstract

**Introduction:**

Autism spectrum disorder is a lifelong neurodevelopmental disorder. The profile of functioning in autistic people is very heterogeneous, and it is necessary to take into account individual characteristics to better support integration in the workplace. However, unemployment rates are higher for autistic people than for other types of disabilities. We present a prospective case series to explore the feasibility and efficacy of an individual-supported program to enhance placement in a sheltered work environment delivered by an Italian community day care center.

**Methods:**

Autistic subjects, aged from 12 to 31 years, participated in an individual-supported program regarding employment in sheltered art workshops, integrated into the regular activity of a semi-residential center three times a week for 1 year. Their feasibility retention rate and time worked per session were registered; moreover, working methods efficacy and self-organization improvement were tracked by the Likert-based rating system. Secondary outcome measures span functional levels, challenge behaviors, and sensory problems.

**Results:**

All the individuals presented a good adaptation to the environment, with a significant increase in time worked per session. After 1 year, the intervention allowed an increase in tasks completed in an assigned complex job and an improvement in self-organization within the work schedule in a group of subjects consisting mainly of severe-to-moderate levels of autism severity (86.6%). Finally, we observed a significant increase in independent functioning areas of the TEACCH transitional assessment profile. Challenge behaviors and sensory problems were also recorded.

**Conclusion:**

This case series supports the idea that individual-supported programs for placement in sheltered job environments delivered by community day care centers could be feasible and effective for ASD with higher levels of severity and co-occurring intellectual disability. Further targeted studies based on community models and accessible methods need to be planned to define the effectiveness of the intervention and promote improved practice at the community level with a better social impact.

## 1. Introduction

Autism spectrum disorder (ASD) is an early onset, pervasive, and lifelong neurodevelopmental disorder (1). After the first diagnosis, psychosocial and behavioral interventions are needed to ameliorate core symptoms and to improve specific skills, such as joint attention, language, and social engagement (2, 3). Thereafter, in adolescence and adulthood, the treatment is focused on social skills (4–6), emotional and behavioral problems (7, 8), managing the co-occurring mental health conditions (9–11) and promoting independence, personal autonomy, and self-employment (12–14). However, many autistic adolescents or young adults do not receive adequate support for attaining and maintaining employment (15). Indeed, over the transition period from school to work, only 25% of autistic individuals got paid work (16). Specifically, 80% of autistic adults are estimated to be jobless worldwide (17), and recent reports affirm that unemployment rates are higher for autistic people than for other types of disabilities (18, 19). Conversely, it has been recorded that people with autism who receive vocational rehabilitation services have a higher rate of competitive employment (37%). Social inclusion labs offer training and dignity to young people with ASD, but it is estimated that only 1.1% have received them, making the unemployment rate higher (20). Therefore, it should be considered the use of integrated vocational rehabilitation and high school employment transition programs, especially some components of these interventions, such as vocational counseling and intensive employment support services (21). Thus, outcomes for autistic adults have frequently been described as poor, although there is variability based on individual characteristics including intelligence quotient (IQ), ASD severity, socio-economic status, and parental support (22). Autistic subjects often experience difficulties with social interactions, problem-solving in social situations, and troubles with changing routines, based on their neuropsychological profile, which could cause problems and loss of work (23). Indeed, the profile of functioning in autistic people is very heterogeneous, and it is necessary to take into account individual characteristics to better support integration in the workplace (24). Often, autistic people show intellectual disability (ID) (25) and psychiatric comorbidities (26–28). A recent study showed that ~31% of 8-year-old children with ASD presented a co-occurring intellectual impairment (intelligence quotient <70) (29). The Centers for Disease Control and Prevention confirmed this percentage and reported that ~33% of autistic children were estimated to have ID (30). Despite this high prevalence, autistic individuals with intellectual disability are often excluded or routinely under-recruited in studies regarding interventions to improve vocational integration with a consequent lack of data (31).

Usually, existing employment options include sheltered employment, supported employment, and competitive employment (32). The majority of the studies have evaluated inclusion programs in sheltered workshops, which allow autistic adults to obtain a job more easily (33). Most of these programs start by identifying the individual's strengths and interests, aiming to find the right job position, and finally providing the necessary support and strategies to improve adjustment, social cognition, and independence (34–36). However, the systematic review of Walsh et al. (37) highlighted the paucity of research that addresses sheltered employment despite the great opportunity that it represents for autistic people. Among the experiences reported in the literature, the Adult Life-Skills Program of the Princeton Child Development Institute (PCDI) begins preparation well before the age of 21 years, and the areas in which intervention is provided concern fundamental skills (care self, language, social skills, leisure skills, community skills, and work skills) (14). Another college-to-career transition intervention program, PEERS^®^ for Careers, has shown promise in addressing the multi-faceted needs of autistic people in the workplace. Autistic pre-adults have been able to learn employment-related social skills (38). However, among the critical issues encountered, difficulties in the social aspects of work are reported, especially in communication with supervisors or colleagues (39).

In Italy, there are several projects for the job placement of autistic people. Most of them provide individual paths based on specific characteristics. The main activities involve farm-related chores (Cascina Rossago, residential farm community, Pavia; European Therapeutic Center, Florence) (40), catering works (A.L.I. Project, Ascot srl, Florence; PizzAut Onlus, Milan), and artistic pathways (Fondazione Bambini e Autismo Onlus, Pordenone). In Modica, where in Sicily, a project was implemented leading to the creation of a B & B run by young adults with neurodiversity, including ASD (La Casa di Toti onlus).

We present a prospective case series that evaluated the feasibility and effectiveness of a 1-year individual-support program for autistic people, both adolescents and adults, in a Musiva Art Laboratory. Within the workshop, autistic people were involved in the production and promotion of artworks. The lab was integrated into an Italian community service, in the province of Messina, in Barcellona Pozzo di Gotto, operated by the Social Coop “Progetto Dopo di Noi.” The principal aim was to improve placement in a sheltered work environment, teaching functional skills and self-directed actions to autistic young adults who have heterogeneous profiles and a wide range of intellectual functioning.

## 2. Materials and methods

### 2.1. Participants and ethics

Subjects were consecutively enrolled and treated at the specialized Autism “Progetto Dopo di Noi” in Barcelona P.G., Italy, and evaluated at the IRCCS Centro Neurolesi “Bonino-Pulejo”, Messina, from January 2021 to January 2023. The local Ethics Committee approved the study (IRCCSME 39/2017). Researchers provided clear and concise study information to most participants even using supportive tools based on each subject's functioning to maximize participation in decision-making. Written informed consent was obtained from participants or from both parents according to the terms established by law. All procedures comply with the ethical standards of the relevant national and institutional committees on human experimentation and with the Helsinki Declaration. The inclusion criteria were fulfilling DSM-5 diagnostic criteria for autism spectrum disorder (1), age between 12 and 35 years old, and minimal communication ability (verbal speech or augmentative and alternative communication, AAC). Participants were excluded if they presented severe aggressive behaviors or severe hypersensitivity to the work materials (i.e., clay, glass, painting, or glue).

### 2.2. Measures and design

At the time of recruitment, all subjects undergo a complete diagnostic assessment including neuropsychological and behavioral testing. IQ was determined using the Leiter International Performance Scale—third edition (41). Autistic behaviors were assessed using the Autism Diagnostic Observation Scales—second edition (ADOS-2) (42). Adaptive functioning was evaluated using the Vineland Adaptive Behavior Scales—second edition (VABS-II) (43).

Feasibility was evaluated in terms of engagement and participation in the educational programs and demonstrated through analysis of service utilization and retention rate. In addition, we measured compliance by registering the time in minutes that each participant worked per session.

To evaluate preliminary efficacy, we documented specific target behaviors through task analysis and data collection methods by two independently trained therapists. The average inter-observer agreement (IOA) across all participants was 93.6%. Specifically, we evaluated (A) work chain procedure (as the number of tasks executed) and (B) self-organization and autonomy in the work processes using both a six-point Likert-based rating system that focuses on specific skills; for more details (see Supplementary Table S1).

Secondary outcome measures included the TEACCH Transitional Assessment Profile (TTAP) (44): the TTAP is designed to provide assessment data for transition planning from school age to adolescence and adulthood, it is a criterion-referenced test designed for autistic individuals who have mild-to-severe intellectual disabilities and are over 12 years of age. We used the Direct Observation Scale of the TTAP in this study. The TTAP test items cover the six functional areas of vocational skills (VS), vocational behaviors (VB), independent functioning (IF), leisure skills, functional communication, and interpersonal behavior. Based on the observation in specific assessment tasks, the psychologist rated participants' performance on a three-point scale consisting of pass (P), emerging (E), or fail. Each scale consists of twelve sub-items, which indicate different functional levels. As outcome measures of the ISP, we take into account only the VS, VB, and IF subscales. Participants were evaluated before (T0) and after (T1) the completion of the intervention program, by the same blinded expert child psychologist and psychiatrist. Furthermore, each subject based on tactile hypersensitivity was gradually exposed to the material used in the art laboratory (clay, paint, or glass); to measure material tolerability, data were taken at the time of manipulation in the absence of problem behaviors or signs of distress. In addition, we measured challenging behaviors (i.e., hetero-directed aggression, self-injurious behavior, or throwing and destroying objects) through the frequency/event and rate recording method, which implicates counting and recording the number of times a behavior happens within each work session by two independent trained therapists (45, 46).

To analyze differences at the two time points, we adopted repeated measures ANOVA, using severity level as a covariate. To account for assumptions of violation of repeated measures ANOVA, we used the Greenhouse–Geisser degrees of freedom correction strategy.

### 2.3. Intervention

The people participated in an individual-supported program regarding employment in sheltered art workshops, integrated into the regular activity of a semi-residential center for autistic adolescents and young adults, three times a week for 1 year. The essential goal of ISP was to increase compliance with work rules and the cooperative work method (work chain). Simultaneous to the professional goals, we aimed to improve independence and self-organization and to develop new skills and their ability to adapt to the work environment.

The standardized ISP was based on the TEACCH approach (34) adapted within the applied behavior-analytic framework; the structure and strategies used for the ISP are elucidated in Supplementary Table S2. The work program was set up after an initial assessment of the functional skills (TTAP) of each participant. Autistic individuals have been included in the laboratory, involving them in the production and promotion of their artistic projects. The work programs were carried out by a qualified group of therapists and art teachers. Each session was organized through a structured environment program based on the TEACCH method, aimed at hiring in the laboratory as employees (47). Environment and individual activities were organized to optimize learning and avoid frustration. Participants, through an individualized program, experienced an increase in structure to promote independence by adult guidance/suggestions from the organization of the physical environment (e.g., settled tables with well-divided materials in the same position, minimizing possible distractions in a manner consistent with the people's needs), activity systems (e.g., predictable arrangement of activities by the use of visual schedules of work routines; basic left-to-right-to-finished routines across work routines), visually structured activities (e.g., materials designed to specifically target or explicitly teach skills), well-established routines (e.g., visual countdown), and positive behavior management (48). In addition, applied behavior analysis (ABA) principles were used, which include different procedures that can be well combined as well as the use of the visual prompt and subsequently of the fading. Furthermore, chaining has been used to promote autonomy and job skill learning: a stimulus-response chain is a sequence of discriminative stimuli (SD) and responses (R) of which each R, except the last, provides the SD for the next ones, while the last one is typically followed by a reinforce (49).

Based on the individual ability of each person, the main method to be used to teach a stimulus–response chain was chosen, namely:

- The entire presentation of the task (through this modality, the subject tries every time all the steps from the beginning to the end of the sequence, only then he will carry out the whole task until he reaches a certain mastery in each step).
- Retrograde chaining (gradually building the chain in the opposite direction to that in which it is normally done; that is, first the last step is established, then the penultimate is taught and concatenated with the last, then the antepenultimate is concatenated with the other two, and so on, continuing back to the beginning of the chain).
- Forward chaining (the initial step in the chain is taught first and then the second step is taught; these are chained together, moving on to the first three steps, and so on until the entire sequence is learned).

## 3. Result

**Figure 1:**
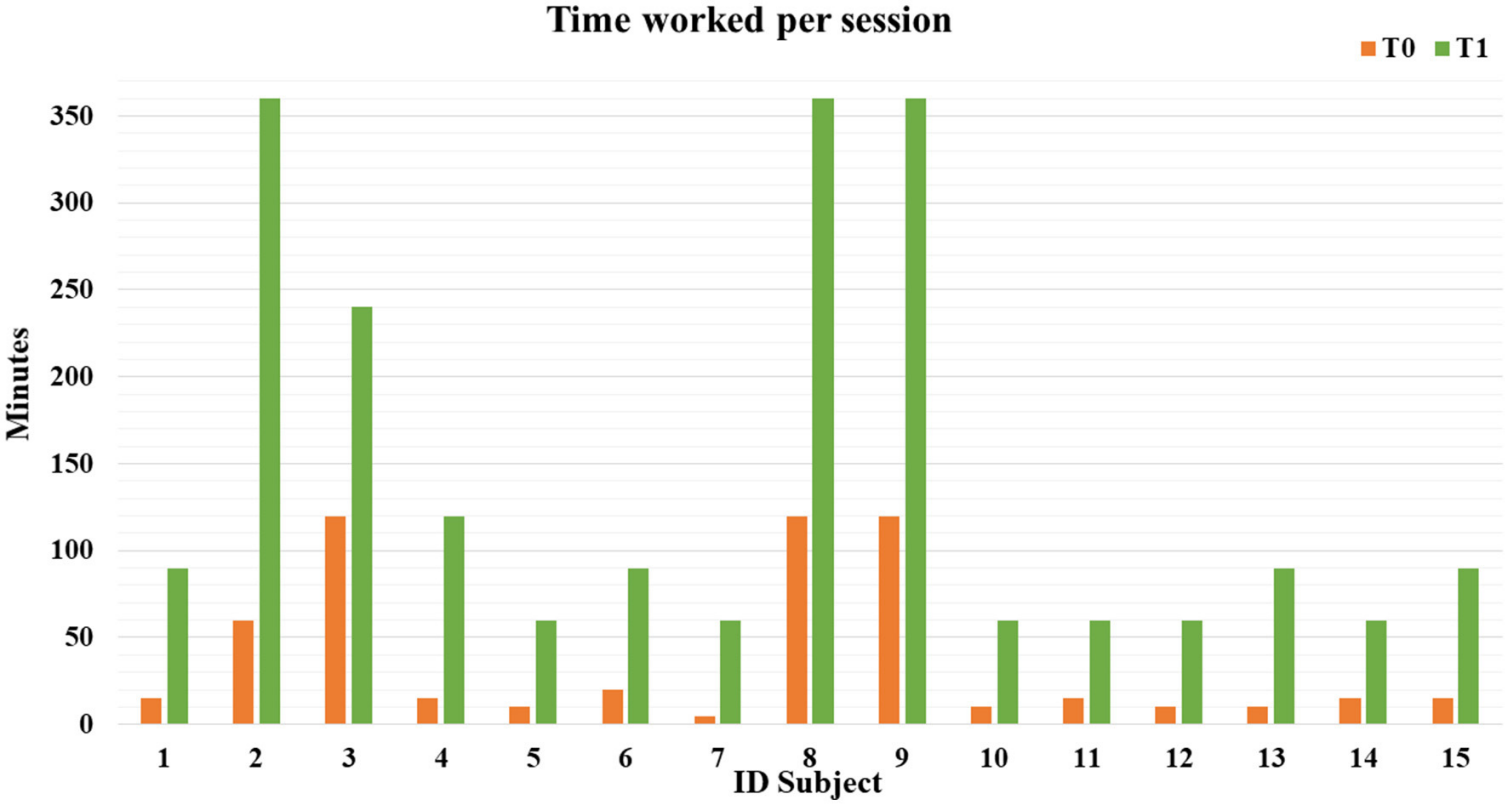
Time worked per session for each subject at T0 (baseline) and T1 (1 year later).

**Figure 2:**
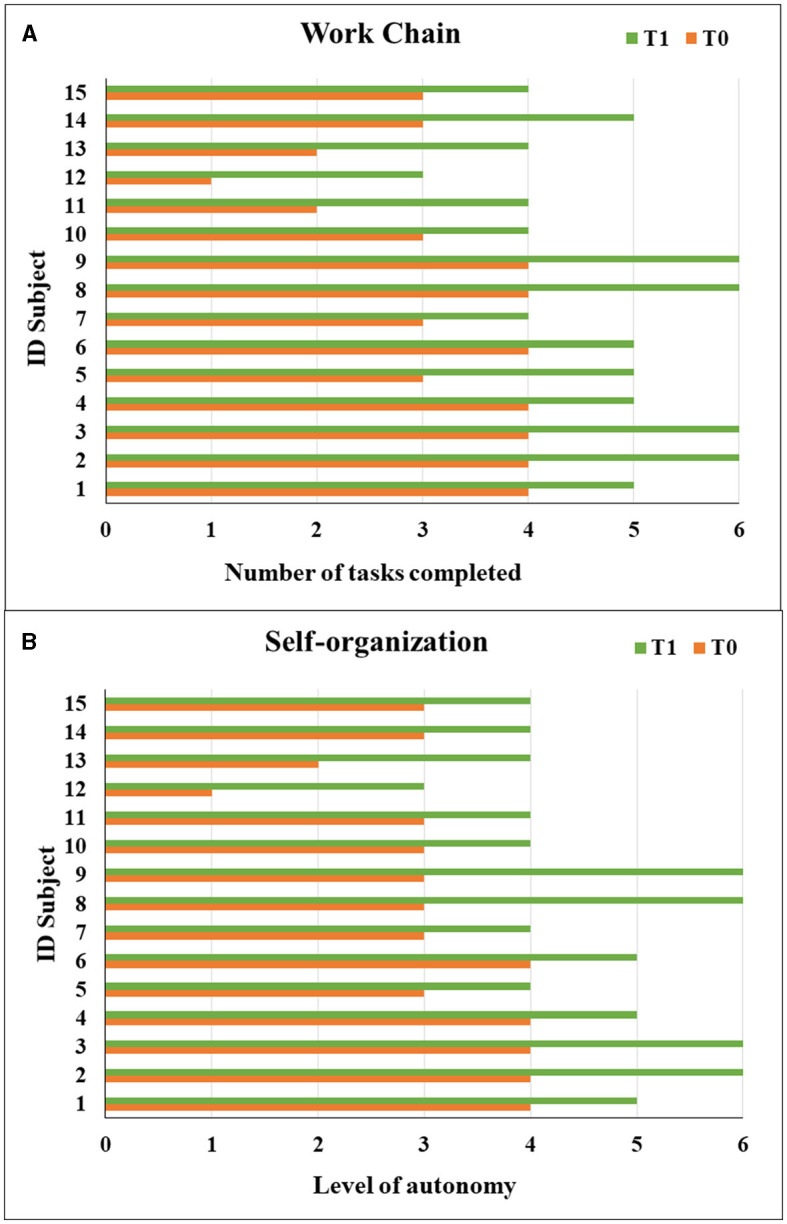
Tasks completed in the work chain procedure from each subject at T0 and T1 **(A)**. Self-organization and independence in work sessions for each subject at T0 and T1 **(B)**.

**Figure 3:**
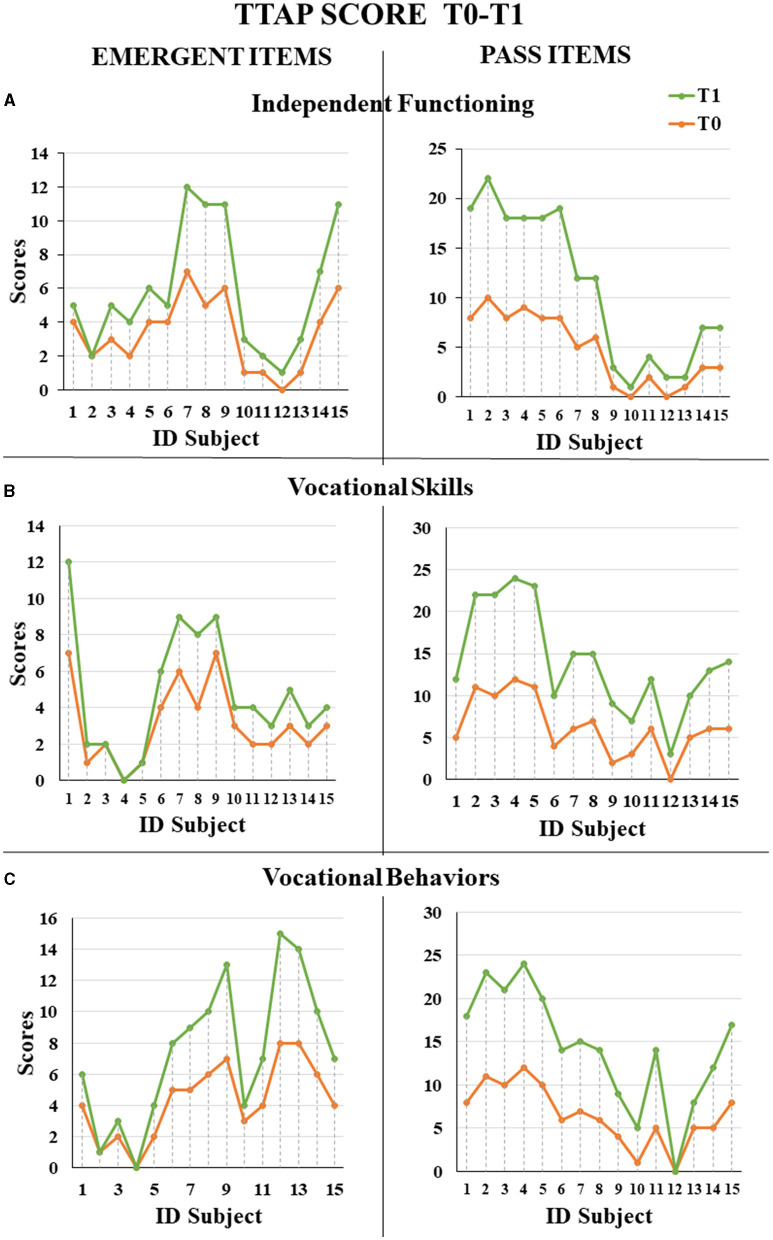
TTAP emergent and pass scores for each subject at T0 and T1 **(A)** Independent functioning scores; **(B)** Vocational skills score; **(C)** Vocational behaviors score.

**Figure 4:**
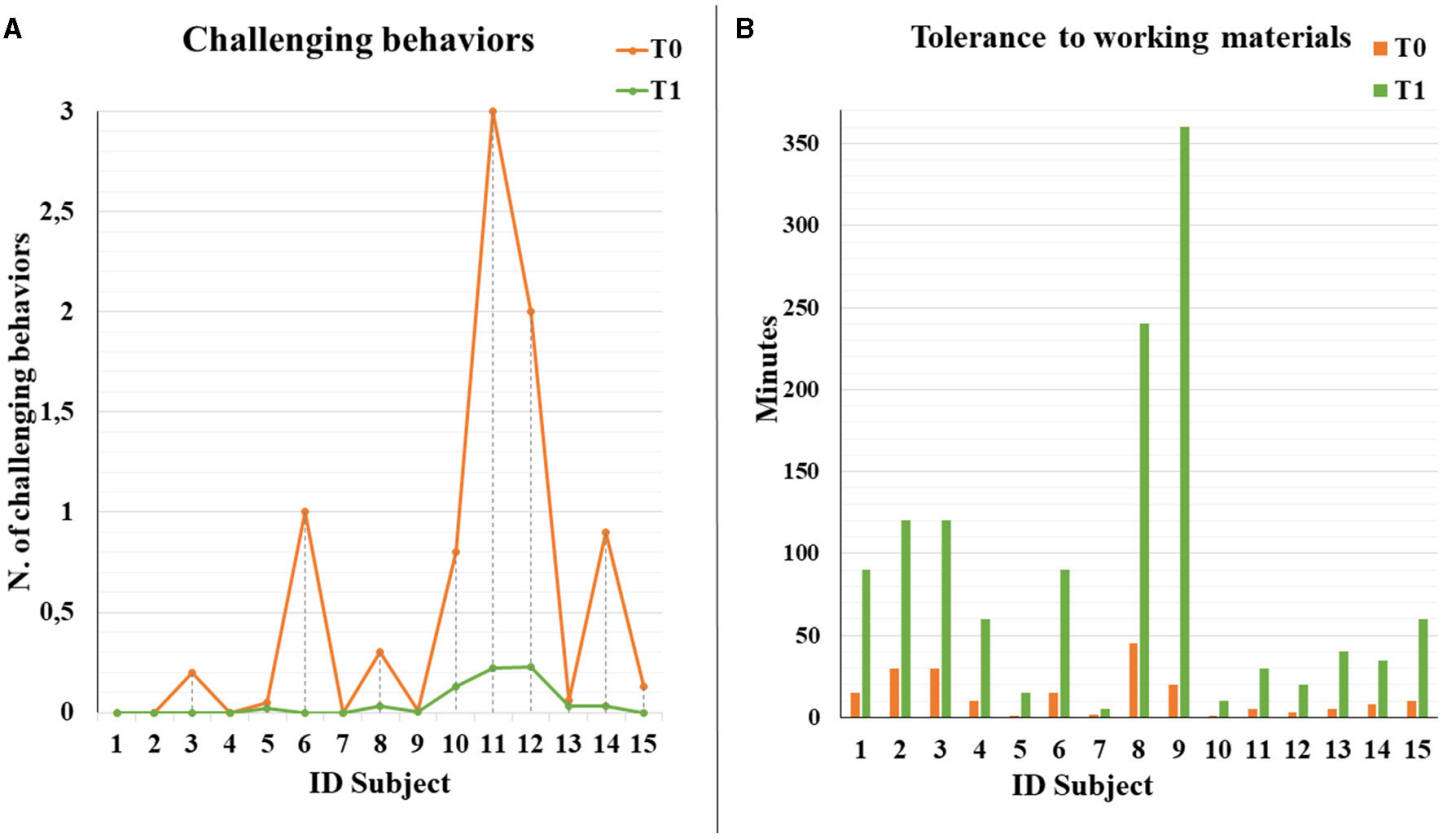
Challenging behavior rates recorded for each subject at T0 and T1 **(A)**. Time spent to work with the material then initially was poorly tolerated for each subject at T0 and T1 **(B)**.

In total, we enrolled fifteen autistic people, aged from 12 to 31 years (with mean age 22.5 ± 5.3 years), with a percentage of adolescent subjects of 21.1%. Three were female subjects, and 12 were male subjects, with M:F = 4:1 ratio. All the subjects fulfilled the autistic cutoff value in ADOS2; among those, eight (53.3%) presented an autism severity level 3, five (33.3%) presented level 2, and two (13.3%) presented level 1, which accompanied intellectual disability (IQ level ≤ 70) in 10 subjects (66.7%). The mean adaptive behavior composite score was 90.1 at VABS-II, see global demographic details in Table 1. Specifically, three individuals (20.0%) displayed severe language impairment, while five (33.3%) and seven (46.7%) individuals had moderate and mild impairment, respectively. Challenging behaviors were exhibited more frequently by seven (46.7%) subjects, and five subjects (33.3%) presented psychiatric comorbidity, represented by obsessive-compulsive disorder (*N* = 3, 60.0%), anxiety disorder (*N* = 1, 20.0%), major depressive disorder (*N* = 1, 20.0%); Supplementary Table S3 shows whole clinical and behavioral features of each person. Regarding engagement and participation, the retention rate of autistic people who completed 1 year of ISP was 100%. All the individuals presented a good adaptation to the environment. Specifically, we found a significant increase in time worked per session (*F* = 4.73, *p* = 0.048; Figure 1). After 1 year of ISP, the number of tasks completed in the work chain procedure increased significantly for all kinds of subjects (*F* = 6.33, *p*= 0.026; Figure 2A); furthermore, the number of tasks completed independently increased although not in a statistically significant way: for most participants, task engagement and self-organization were maintained even without the job coach's control (Figure 2B). During the work period in the art lab, some individuals showed such involvement that they were able to create entire works of art by themselves (Supplementary Figure S1). Moreover, we analyzed the passed items (P) in TTAP functional areas of vocational skills, vocational behaviors, and independent functioning. Among those, by repeated measures-ANOVA, we observed a significant increase in the IF subscale at T1 (*F* = 22.29, *p* < 0.001; Figure 3A) as well as significant interaction with the severity level (*F* = 9.43, *p* = 0.009). The same trend was verified in IF emerging items too (*F* = 21.41, *p* < 0.001) as well as significant interaction with severity level (*F* = 15.29, *p* = 0.002). There were no significant improvements in the VS and VB areas (Figures 3B, C). At baseline, most subjects showed mild tactile hypersensitivity to manipulation. During the art lab, each subject was gradually exposed to and worked with different materials (clay, glass, paint, and glue) that had different sensory characteristics. In particular, eight subjects (ID 1, 2, 3, 4, 6, 8, 9, and 15) presented a great improvement and were then able to manipulate the material for a longer time, without presenting signs of stress (Figure 4). Finally, 11 subjects presented problematic behaviors at the beginning of ISP, reduced over time or completely resolved for three individuals.

**Table 1:** Demographic and clinical characteristics of the entire sample.

	***N***	**Mean ±SEM or %**	**Range**
Age in years	*N* = 15	22.5 ± 5.3	12–31
**Sex**			
Male	12	80%	
Female	3	20%	
M:F ratio	4:1		
**ASD severity**			
Level 1	2	13.3%	
Level 2	5	33.3%	
Level 3	8	53.3%	
**I.Q**.			
>70	5	33.3%	
≤ 70	10	66.7%	
**VABS-II score**			
Communication	15	22.25	20–29
Daily living skills	15	28.75	20–39
Socialization	15	22	20–28
Adaptive behavior composite score	15	90.1	50–153
**ADOS-2**			
On the autism spectrum	0	0%	
Autistic	15	100%	

IQ, intelligence quotient; VABS-II, Vineland Adaptive Behavior Scales II edition; ADOS-2, Autism Diagnostic Observation Schedule-second edition.

## 4. Conclusion

In this prospective observational case series, we described 15 autistic individuals who participated in an individual-support program integrated into the rehabilitative activities of an Italian semi-residential center for autistic adolescents and young adults. The group consisted of ASDs of different severity, with a greater presence of moderate and severe levels; furthermore, 66.7% of them presented a cognitive disability, accompanied by impairment in adaptive function. The ISP aimed to teach functional skills and self-directed actions usefully to employment in sheltered art workshops. Established on individual needs, the ISP program was based on the TEACCH approach in combination with applied behavior-analytic principles. This study mainly focused on (a) feasibility and engagement documented through retention rate and time in minutes that each participant worked per session; (b) working methods efficacy and self-organization improvement tracked by the Likert-based rating system; (c) changes in the level of functioning through TTAP (VS, VB, and IF areas); (d) management of challenging behaviors by registered the frequency/event and rate recording method; and (e) tolerance to tactile sensory stimuli through the time in minutes of material manipulation in the absence of signs of distress. Collectively, this case series demonstrated the feasibility of integrating professional ISP into a semi-residential Italian center, documented by the full participation in the educational programs, and the growing time in minutes that each participant worked per session. Moreover, the intervention allowed an increase of tasks completed in an assigned complex job and an improvement in self-organization and autonomy within the work schedule in a group of people consisting mainly of severe-to-moderate levels of autism severity (86.6%). This achievement was accompanied by an improvement in the IF areas, both in passed and emergent items.

By looking at the improvement trend, even though the sample size is too small to draw absolute conclusions, the time worked per session and the tolerance to work materials appear to be the variables that, at baseline, guide a better result and a greater enhancement, independently by the autism severity level and the intellectual functioning (Supplementary Figure S2). These considerations may suggest what has already been stated in the literature: a greater correspondence between professional aptitude and occupational placement may improve engagement, learning, and behavioral development (50, 51). The degree of intellectual disability influences outcomes in adulthood (52), but it is clear that this is not the only factor, and the impact of other variables, including personal preference, needs to be evaluated (37). Although these results cannot be generally applicable, this report supports the idea that individual-supported programs could be effective in preparing autistic subjects with higher levels of severity and co-occurring intellectual disability for placement in sheltered job environments, in line with previous studies (53). It will be favorable to consider it as part of the usual activities planned by daily community rehabilitation centers, especially since it should be designed for the most levels of ASD severity and intellectual disability to increase skills or vocational dispositions that will help them in adaptive functioning and increase their community participation (54–56). It is also useful to promote the job placement of adults with autism, with or without intellectual disabilities, to organize comprehensible timed sequential programs that promote choice and autonomy, and an individualized work schedule and a well-defined role must be considered, to provide participants with the necessary adjustment phase (57). Many autistic people display unique skills that may be valuable to specific employment, such as attention to detail and tolerance for repetition (58, 59). The feasibility of an individual-supported program to enhance placement in a sheltered work environment in the Italian community is supported by other experiences on the national territory. The implementation of publicly funded employment services is critical in view of the rapid increase of adults with autism in need of employment support. A recent study conducted a survey for autistic adults to improve the understanding of current services experienced in different countries of the European Union (60). The responders globally preferred autism-specific support services; however, autistic adults living in Italy reported more often that they had tried to get a service and failed (residential, employment, and education services). It seems worthwhile to suggest that the Italian Public Health System continue to allocate resources for the training of health professionals and to promote and facilitate greater community involvement for autistic people, including through integrated vocational pathways in daycare centers. One of the main limitations of this study is the number of subjects reported, therefore, further studies with bigger sample sizes have to be conducted in order to corroborate our results. Thus, this small sample cannot be representative of the ASD population; however, including all consecutive autistic individuals reduces the chances of bias and increases the validity for generalizing the results. In addition, the sample size in our study is relatively small in statistical terms, which limits the conclusions that can be drawn from these data. A second important limitation was that we only measured participation and engagement in educational programs, in terms of service usage and retention rate. We missed an important occasion to collect and take into account participants' and their parents' point of view in a standardized way. Gathering different perspectives who share their lived experiences of services is very important in developing a service (61); it would have been desirable to devise a more participatory approach from the outset, with the inclusion of the autistic perspective (62). Finally, we did not have a control group to compare outcomes and feasibility. However, this would be a beginning step: we believe that these case series could help health professionals to stake on their individuals, and, given our results, it seems useful to propose that the rehabilitation centers and semi-residential services plan individual-supported programs to enhance placement in a sheltered work environment for qualifying inclusive and personalized emancipation paths of autistic young. In moving forward, it seems necessary that the social inclusion efforts favor the maturation and growth of the individual through qualified and specific paths of development of skills that allow to achieve a degree of personal, social, and work autonomy, appropriate to everyone's potential (63). There is a need to identify better strategies to guide the choice of goals within the adolescent transition (64) and to build a better exchange of community and work-place supports: adults with disabilities often aspire to start and maintain real jobs (65), and an individual-supported program may offer the means for achieving this goal. Research related to transition and employment in autism needs more development, filling the gap of evidence-informed approaches guiding service delivery (66). Further research is needed to define how to address the heterogeneity of ASD concerning professional support and to better clarify the key elements of community-based support models related to autistic individuals in adulthood. In addition to community studies on the short-term work of autistic people, it would also be appropriate to implement research on subjects in need of long-term work services, identifying the favorable prognostic neuropsychological characteristics.

## Data availability statement

The raw data supporting the conclusions of this article will be made available by the authors, without undue reservation.

## Ethics statement

The studies involving humans were approved by Comitato Etico IRCCS Sicilia—Sezione Centro Neurolesi Bonino-Pulejo. The studies were conducted in accordance with the local legislation and institutional requirements. Written informed consent for participation in this study was provided by the participants' legal guardians/next of kin.

## Author contributions

RM, LT, and CC participated in the study design and coordination, provided clinical oversight for data collection and interpretation, and drafted the manuscript. AQ and FC conceptualized the study and supervised the manuscript. MDC participated in data collection and processing, contributed to the literature review, and helped draft the manuscript. FC performed statistical analysis of the data. ET and CI contributed to the literature review and helped draft the manuscript. CP supervised the coordination of the study. All authors have read and agreed to the published version of the manuscript.

## References

[1] American Psychiatric Association. Diagnostic and Statistical Manual of Mental Disorders, 5th ed. Washington, DC: American Psychiatric Association (2013). 10.1176/appi.books.9780890425596

[2] Fuller EA Oliver K Vejnoska SF Rogers SJ. The effects of the early start Denver model for children with autism spectrum disorder: a meta-analysis. Brain Sci. (2020) 10:368. 10.3390/brainsci10060368 32545615 PMC7349854

[3] Reichow B Hume K Barton EE Boyd BA. Early intensive behavioral intervention (EIBI) for young children with autism spectrum disorders (ASD). Cochrane Database Syst Rev. (2018) 5:CD009260. 10.1002/14651858.CD009260.pub3 29742275 PMC6494600

[4] Laugeson EA Frankel F Gantman A Dillon AR Mogil C. Evidence-based social skills training for adolescents with autism spectrum disorders: the UCLA PEERS program. J Autism Dev Disord. (2012) 42:1025–36. 10.1007/s10803-011-1339-1 21858588

[5] Reichow B Servili C Yasamy MT Barbui C Saxena S. Non-specialist psychosocial interventions for children and adolescents with intellectual disability or lower-functioning autism spectrum disorders: a systematic review. PLoS Med. (2013) 10:e1001572. 10.1371/journal.pmed.1001572 24358029 PMC3866092

[6] Laugeson EA Frankel F. Social Skills for Teenagers with Developmental and Autism Spectrum Disorders: The PEERS Treatment Manual. London: Routledge (2011). 10.4324/9780203867686 25860311

[7] Tarver J Palmer M Webb S Scott S Slonims V Simonoff E . Child and parent outcomes following parent interventions for child emotional and behavioral problems in autism spectrum disorders: a systematic review and meta-analysis. Autism. (2019) 23:1630–44. 10.1177/1362361319830042 30818965

[8] Bearss K Johnson C Smith T Lecavalier L Swiezy N Aman M . Effect of parent training vs parent education on behavioral problems in children with autism spectrum disorder: a randomized clinical trial. JAMA. (2015) 313:1524–33. 10.1001/jama.2015.3150 25898050 PMC9078140

[9] Hume K Steinbrenner J. R Odom S. L Morin K. L Nowell S. W Tomaszewski B . Evidence-based practices for children, youth, and young adults with autism: third generation review. J Autism Dev Disord. (2021) 51:4013–32. 10.1007/s10803-020-04844-2 33449225 PMC8510990

[10] Keefer A Kreiser NL Singh V Blakeley-Smith A Reaven J Vasa RA. Exploring relationships between negative cognitions and anxiety symptoms in youth with autism spectrum disorder. Behav Ther. (2018) 49:730–40. 10.1016/j.beth.2017.12.002 30146140

[11] Linden A Best L Elise F Roberts D Branagan A Tay YBE . Benefits and harms of interventions to improve anxiety, depression, and other mental health outcomes for autistic people: a systematic review and network meta-analysis of randomised controlled trials. Autism. (2023) 27:7–30. 10.1177/13623613221117931 35957523 PMC9806485

[12] Lord C Brugha TS Charman T Cusack J Dumas G Frazier T . Autism spectrum disorder. Nature Rev Dis Primers. (2020) 6:5. 10.1038/s41572-019-0138-4 31949163 PMC8900942

[13] Bishop-Fitzpatrick L Minshew NJ Eack SM. A systematic review of psychosocial interventions for adults with autism spectrum disorders. J Autism Dev Disord. (2014) 43:687–94. 10.1007/s10803-012-1615-8 22825929 PMC3508309

[14] McClannahan LE MacDuff GS Krantz PJ. Behavior analysis and intervention for adults with autism. Behav Modif. (2002) 26:9–26. 10.1177/0145445502026001002 11799656

[15] Shattuck PT Wagner M Narendorf S Sterzing P Hensley M. Post-high school service use among young adults with an autism spectrum disorder. Arch Pediatr Adolesc Med. (2011) 165:141–6. 10.1001/archpediatrics.2010.279 21300654 PMC3097532

[16] Roux AM Garfeld T Shattuck PT. Employment policy and autism: analysis of state Workforce Innovation and Opportunity Act (WIOA) implementation plans. J Vocat Rehabil. (2019) 51:285–98. 10.3233/JVR-191046

[17] Ki-moon B. Secretary-general Invites Business to Commit to Hiring People with Autism, as he Launches ‘Call to Action' Initiative on World Day (2015). Retrieved June (2015) 8:2018.

[18] Sparkes I Riley E Cook B Machuel P. Office for National Statistics. Outcomes for Disabled People in the UK: 2020 (2021). Available online at: https://www.ons.gov.uk/peoplepopulationandcommunity/healthandsocialcare/disability/articles/outcomesfordisabledpeopleintheuk/2021

[19] Shattuck PT Narendorf SC Cooper B Sterzing PR Wagner M Taylor JL. (2012). Postsecondary education and employment among youth with an autism spectrum disorder. Pediatrics. (2012) 129:1042–9. 10.1542/peds.2011-2864 22585766 PMC3362908

[20] Roux AM Miller KK Tao S Rast JE Ventimiglia J Shattuck PT . Unrealized cross-system opportunities to improve employment and employment-related services among autistic individuals. Milbank Q. (2023) 37526044 10.1111/1468-0009.12666 37526044 PMC10726849

[21] Schall C Wehman P Avellone L Taylor JP. Competitive integrated employment for youth and adults with autism: findings from a scoping review. Child Adolesc Psychiatr Clin. (2020) 29:373–97. 10.1016/j.chc.2019.12.001 32169268

[22] Scott M Milbourn B Falkmer M Black M Bölte S Halladay A . Factors impacting employment for people with autism spectrum disorder: a scoping review. Autism. (2019) 23:869–901. 10.1177/1362361318787789 30073870

[23] Lindsay S Osten V Rezai M Bui S. Disclosure and workplace accommodations for people with autism: a systematic review. Disabil Rehabil. (2021) 43:597–610. 10.1080/09638288.2019.1635658 31282214

[24] Müller E Schuler A Burton BA Yates GB. Meeting the vocational support needs of individuals with Asperger syndrome and other autism spectrum disabilities. J Vocat Rehabil. (2003) 18:163–75.

[25] Fombonne E. Epidemiology of autistic disorder and other pervasive developmental disorders. J Clin Psychiatry. (2005) 66:3. 10.1002/9780470939345.ch2 16401144

[26] Van Steensel FJ Bogels SM Perrin S. Anxiety disorders in children and adolescents with autistic spectrum disorders: a meta-analysis. Clin Child Fam Psychol Rev. (2011) 14:302–17. 10.1007/s10567-011-0097-0 21735077 PMC3162631

[27] Risi S Lord C Gotham K Corsello C Chrysler C Szatmari P . Combining information from multiple sources in the diagnosis of autism spectrum disorders. J Am Acad Child Adolesc Psychiatry. (2006) 45:1094–103. 10.1097/01.chi.0000227880.42780.0e 16926617

[28] Persico AM Cucinotta F Ricciardello A Turriziani L Chen B. Chapter 3: Autisms. Comprehensive developmental neuroscience. Neurodev Disord. (2020) 35–77. 10.1016/B978-0-12-814409-1.00003-3

[29] Shenouda J Barrett E Davidow AL Sidwell K Lescott C Halperin W . Prevalence and disparities in the detection of autism without intellectual disability. Pediatrics. (2023) 151:e2022056594. 10.1542/peds.2022-056594 36700335

[30] Zeidan J Fombonne E Scorah J Ibrahim A Durkin MS Saxena S . Global prevalence of autism: a systematic review update. Autism Res. (2022) 15:778–90. 10.1002/aur.2696 35238171 PMC9310578

[31] Russell G Mandy W Elliott D White R Pittwood T Ford T. Selection bias on intellectual ability in autism research: a cross-sectional review and meta-analysis. Mol Autism. (2019) 10:1–10. 10.1186/s13229-019-0260-x 30867896 PMC6397505

[32] Gottlieb A Myhill WN Blanck P. Employment of people with disabilities. International Encyclopedia of Rehabilitation. (2010).

[33] Taylor JL Seltzer MM. Employment and post-secondary educational activities for young adults with autism spectrum disorders during the transition to adulthood. J Autism Dev Disord. (2011) 41:566–74. 10.1007/s10803-010-1070-3 20640591 PMC3033449

[34] Keel JH Mesibov GB Woods AV. TEACCH-supported employment program. J Autism Dev Disord. (1997) 27:3–9. 10.1023/A:1025813020229 9018578

[35] Howlin P. Outcomes in autism spectrum disorders. In:Volkmar FR Paul R Klin A Cohen D, editors. Handbook of Autism and Pervasive Developmental Disorders, Volume 1. Hoboken, NJ: John Wiley & Sons, Inc. (2005) 1:201–20. 10.1002/9780470939345.ch7

[36] Mesibov GB Shea V Schopler E. The TEACCH Approach to Autism Spectrum Disorders. New York, NY: Springer Science & Business Media. (2005). 10.1007/978-0-306-48647-0

[37] Walsh L Lydon S Healy O. Employment and vocational skills among individuals with autism spectrum disorder: predictors, impact, and interventions. Rev J Autism Dev Disord. (2014) 1:266–75. 10.1007/s40489-014-0024-7

[38] Moody CT Factor RS Gulsrud AC Grantz CJ Tsai K Jolliffe M . A pilot study of PEERS^®^ for careers: a comprehensive employment-focused social skills intervention for autistic young adults in the United States. Res Dev Disabil. (2022) 128:104287. 10.1016/j.ridd.2022.104287 35772303

[39] Hendricks D. Employment and adults with autism spectrum disorders: challenges and strategies for success. J Vocat Rehabil. (2010) 32:125–34. 10.3233/JVR-2010-0502

[40] Fusar-Poli L Brondino N Orsi P Provenzani U De Micheli A Ucelli di Nemi S . Long-term outcome of a cohort of adults with autism and intellectual disability: a pilot prospective study. Res Dev Disabil. (2017) 60:223–31. 10.1016/j.ridd.2016.10.014 27838208

[41] Roid G Miller L Pomplun M Koch C. Leiter International Performance Scale, 3rd ed. Wood Dale, IL: Stoelting (2013).

[42] Lord C Rutter M DiLavore PC Risi S Gotham K Bishop S. Autism Diagnostic Observation Scales-−2nd edition (ADOS-2). Torrance, CA: Western Psychological Services (2012).

[43] Sparrow SS Cicchetti DV Balla DA. Vineland Adaptive Behavior Scales: Second edition (Vineland II), Survey Interview Form/Caregiver Rating Form. Coushatta, LA: Pearson Assessments (2005). 10.1037/t15164-000

[44] Mesibov G Thomas JB Chapman SM Schopler E. TEACCH Transition Assessment Profile, 2nd ed. Austin, TX: Pro-ed (2007).

[45] Inoue M. Assessments and interventions to address challenging behavior in individuals with intellectual disability and autism spectrum disorder in Japan: a consolidated review. Yonago Acta Med. (2019) 62:169–81. 10.33160/yam.2019.06.001 31320821 PMC6584262

[46] Gardenier NC MacDonald R Green G. Comparison of direct observational methods for measuring stereotypic behavior in children with autism spectrum disorders. Res Dev Disabil. (2004) 25:99–118. 10.1016/j.ridd.2003.05.004 15026089

[47] Siu AMH Lin Z Chung J. An evaluation of the TEACCH approach for teaching functional skills to adults with autism spectrum disorders and intellectual disabilities. Res Dev Disabil. (2019) 90:14–21. 10.1016/j.ridd.2019.04.006 31028977

[48] Van Bourgondien ME Reichle NC Schopler E. Effects of a model treatment approach on adults with autism. J Autism Dev Disord. (2003) 33:131–40. 10.1023/A:1022931224934 12757352

[49] Friman PC. Cooper, Heron, and Heward's applied behavior analysis: checkered flag for students and professors, yellow flag for the field. J Appl Behav Anal. (2010) 43:161–74. 10.1901/jaba.2010.43-161

[50] Taylor JL Smith LE Mailick MR. Engagement in vocational activities promotes behavioral development for adults with autism spectrum disorders. J Autism Dev Disord. (2014) 44:1447–60. 10.1007/s10803-013-2010-9 24287880 PMC4024367

[51] LaRue RH Maraventano JC Budge JL Frischmann T. Matching vocational aptitude and employment choice for adolescents and adults with ASD. Behav Anal Pract. (2019) 13:618–30. 10.1007/s40617-019-00398-7 32953390 PMC7471240

[52] McCauley JB Pickles A Huerta M Lord C. Defining positive outcomes in more and less cognitively able autistic adults. Autism Res. (2020) 13:1548–60. 10.1002/aur.2359 32851813

[53] Hedley D Uljarević M Cameron L Halder S Richdale A Dissanayake C. Employment programmes and interventions targeting adults with autism spectrum disorder: a systematic review of the literature. Autism. (2017) 21:929–41. 10.1177/1362361316661855 27542395

[54] National Institute for Health Care Excellence (NICE). Autism Spectrum Disorder in Adults: Diagnosis and Management. (2012). Available online at: https://www.nice.org.uk/guidance/cg142 (accessed June 14, 2021). 32186834

[55] National Institute for Health and Care Excellence (NICE). Autism Spectrum Disorder in Adults: Diagnosis and Management. London (2021). ISBN-13:978-1-4731-2039-6. 32186834

[56] Advisory Committee on Increasing Competitive Integrated Employment for Individuals with Disabilities Final Report. (2016). Final Report. Available online at: https://www.dol.gov/odep/topics/pdf/ACICIEID_Final_Report_9-8-16.pdf (accessed September 15, 2016).

[57] García-Villamisar D Hughes C. Supported employment improves cognitive performance in adults with autism. J Intellect Disabil Res. (2007) 51(Pt 2):142–50. 10.1111/j.1365-2788.2006.00854.x 17217478

[58] Cope R Remington A. The strengths and abilities of autistic people in the workplace. Autism Adulthood. (2022) 4:22–31. 10.1089/aut.2021.0037 36605563 PMC8992926

[59] Russell G Kapp SK Elliott D Elphick C Gwernan-Jones R Owens C. Mapping the autistic advantage from the accounts of adults diagnosed with autism: a qualitative study. Autism Adulthood. (2019) 1:124–33. 10.1089/aut.2018.0035 31058260 PMC6493410

[60] Micai M Fulceri F Salvitti T Romano G Poustka L Diehm R . Autistic adult services availability, preferences, and user experiences: results from the autism spectrum disorder in the European Union Survey. Front Psychiatry. (2022) 13:919234. 10.3389/fpsyt.2022.919234 35757227 PMC9226363

[61] Burke M Taylor JL. To better meet the needs of autistic people, we need to rethink how we measure services. Autism. (2023) 27:873–5. 10.1177/13623613231164495 37052345 PMC10263256

[62] Pukki H Bettin J Outlaw AG Hennessy J Brook K Dekker M . Autistic Perspectives on the Future of Clinical Autism Research. Autism Adulthood. (2022) 4:93–101. 10.1089/aut.2022.0017 36601072 PMC9242721

[63] Revell G Inge KJ Mank D Wehman P. The Impact of Supported Employment for People with Significant Disabilities: Preliminary Findings from the National Supported Employment Consortium. Virginia Commonwealth University. School of Education. Rehabilitation Research and Training Center, National Supported Employment Consortium (U.S.). Edit Virginia Commonwealth University Rehabilitation Research & Training Center on Workplace Supports. University of Illinois at Urbana-Champaign (1999).

[64] Cavagnola R Alzani L Carnevali D Chiodelli G Corti S Fioriti F . Neurodevelopmental disorders and development of project of life in a lifespan perspective: between habilitation and quality of life. Ann Ist Super Sanita. (2020) 56:230–40. 10.4415/ANN_20_02_13 32567573

[65] Garcia-Villamisar D Wehman P Navarro M. Changes in the quality of autistic people's life that work in supported and sheltered employment. A 5 years follow-up study. J Vocat Rehabil. (2002) 17:309–12.

[66] Nicholas DB Hodgetts S Zwaigenbaum L Smith LE Shattuck P Parr JR . Research needs and priorities for transition and employment in autism: considerations reflected in a “Special Interest Group” at the International Meeting for Autism Research. Autism Res. (2017) 10:15–24. 10.1002/aur.1683 27753278

