# Supplementary material for: An individual-supported program to enhance placement in a sheltered work environment of autistic individuals mostly with intellectual disability: a prospective observational case series in an Italian community service
Source: Front Psychiatry. 2023 Nov 2;14:1225236. doi: 10.3389/fpsyt.2023.1225236 (PMC10651717; doi:10.3389/fpsyt.2023.1225236)
Supplement: Supplementary file 1 [file Table_1.docx]

Supplementary Material

An individual-supported program to enhance sheltered placement in a work environment of autistic individuals mostly with intellectual disability: a prospective observational case series in an Italian community service.

**Roberta Maggio^1^**†**, Laura Turriziani^1,2^**†**, Caterina Campestre^1^, Marcella Di Cara^3^, Emanuela Tripodi^3^, Caterina Impallomeni^3^, Angelo Quartarone^3^, Claudio Passantino^1^, Francesca Cucinotta^3^***

**Supplementary Table S1.** Likert-based rating system focused on (A) work chain and (B) self-organization and autonomy degree in the work processes.

| 1. The **work chain** of each main activity was divided into 6 tasks: | | | | | | | | | | |
| --- | --- | --- | --- | --- | --- | --- | --- | --- | --- | --- |
| **Activity** | **Task 1** | **Task 2** | | **Task 3** | | **Task 4** | | **Task 5** | | **Task 6** |
| **Create objects with clay** | Take a piece of clay | Knead the clay | | Put the clay inside the bars | | Roll out with a rolling pin | | Draw a shape | | Peel the shape out of the clay |
| **Mosaic** | Take a mosaic tile | Take the pincer | | Cut the mosaic tile with pincer | | Take the glue | | Take the brush | | Glue the mosaic tile |
| **Painting** | Take the brush | Take the painting | | Open the painting jar | | Take the right amount of paint with the brush | | Spread the paint in the center | | Spread the paint along the edges |
| 1. **Self-organization and autonomy degree in the work processes**: | | | | | | | | | | |
| **1** | **2** | | **3** | | **4** | | **5** | | **6** | |
| Physical Guidance | Gestural Indication | | Imitation | | Verbal Suggestion | | Supervision | | Automatically Executes | |
