# Supplementary material for: An individual-supported program to enhance placement in a sheltered work environment of autistic individuals mostly with intellectual disability: a prospective observational case series in an Italian community service
Source: Front Psychiatry. 2023 Nov 2;14:1225236. doi: 10.3389/fpsyt.2023.1225236 (PMC10651717; doi:10.3389/fpsyt.2023.1225236)
Supplement: Supplementary file 2 [file Table_2.docx]

Supplementary Material

An individual-supported program to enhance sheltered placement in a work environment of autistic individuals mostly with intellectual disability: a prospective observational case series in an Italian community service.

**Roberta Maggio^1^**†**, Laura Turriziani^1,2^**†**, Caterina Campestre^1^, Marcella Di Cara^3^, Emanuela Tripodi^3^, Caterina Impallomeni^3^, Angelo Quartarone^3^, Claudio Passantino^1^, Francesca Cucinotta^3^***

**Supplementary Table S2.** Structure and Strategies used for ISP program

In the ISP program, we used the follow natural/contextual approaches in addition to TEACCH structuring strategies to promote and achieve engagement and independence.

| **Organization of the physical structure** | Specific setting useful to give predictability and greater understanding of the activity to be performed (each specific area is identified by a unique symbol that distinguish the activity to be performed, e.g. card image, painting image) |
| --- | --- |
| **Visual schedule** | Includes the use of objects and/or pictograms/drawings to the use of daily work journals to move from one activity to another and to define the tasks to be done. |
| **Activity Systems** | The left-to-right working system is used. This system clarifies the working time and when the activity will be finished (eg cutting simple clay shapes/ painting rods/ mosaic – the material used must already be ready and prepared – the reinforcement is delivered at the end of the task). |
| **Modeling** | The modeling technique involves offering of learning experiences through observing the behavior of a subject who acts as a model (in this case, the operator who shows the action to be performed). |
| **Chaining** | Different procedures for teaching behavioral chains. |
| **Fading** | Stimulus control transfer procedure (stimulus characteristics can be faded in or out) |
