# Supplementary material for: An individual-supported program to enhance placement in a sheltered work environment of autistic individuals mostly with intellectual disability: a prospective observational case series in an Italian community service
Source: Front Psychiatry. 2023 Nov 2;14:1225236. doi: 10.3389/fpsyt.2023.1225236 (PMC10651717; doi:10.3389/fpsyt.2023.1225236)
Supplement: Supplementary file 3 [file Table_3.docx]

Supplementary Material

An individual-supported program to enhance sheltered placement in a work environment of autistic individuals mostly with intellectual disability: a prospective observational case series in an Italian community service.

**Roberta Maggio^1^**†**, Laura Turriziani^1,2^**†**, Caterina Campestre^1^, Marcella Di Cara^3^, Emanuela Tripodi^3^, Caterina Impallomeni^3^, Angelo Quartarone^3^, Claudio Passantino^1^, Francesca Cucinotta^3^***

**Supplementary Table S3.** Detailed clinical and behavioral features of each patient

|  | Sex | Age (yy) | Social Communication | Restricted, repetitive behaviors | Autism Severity | Language impairment | Intellectual impairment | Challenging Behaviors | Psychiatric Comorbidity |
| --- | --- | --- | --- | --- | --- | --- | --- | --- | --- |
| Case 1 | M | 12 | Difficulties adjusting behavior to suit various social contexts | Restricted interests | Level 1 | Mild  (functional language) | Absent  (IQ=79) | No | No |
| Case 2 | M | 24 | Limited interaction | Body swings | Level 1 | Mild  (functional language) | Absent  (IQ=84) | No | Major depressive disorder |
| Case 3 | F | 26 | Abnormal social overtures | Restricted interests | Level 2 | Mild  (functional language) | Present  (IQ=64) | No | No |
| Case 4 | M | 21 | Limited initiation of social interaction | Motor  stereotypies | Level 2 | Mild  (functional language) | Absent  (IQ=79) | Mild  hetero-aggressivity | No |
| Case 5 | F | 28 | Interaction is limited to narrow special interests | Body swings | Level 2 | Mild  (functional language) | Absent  (IQ=74) | Mild  hetero-aggressivity | No |
| Case 6 | M | 14 | No eye contact, interaction is limited to narrow special interests | Echolalia, body swings,  hand clapping | Level 2 | Mild  (functional language) | Present  (IQ=69) | No | No |
| Case 7 | M | 24 | Abnormal social overtures | Hand flapping | Level 2 | Mild  (functional language) | Present  (IQ=47) | No | No |
| Case 8 | M | 25 | Makes unusual approaches to meet needs | Strolling, echolalia,  spitting saliva | Level 3 | Moderate  (few sentences  echolalia) | Absent  (IQ=72) | Mild  hetero-aggressivity | OCD |
| Case 9 | M | 31 | Makes unusual approaches to meet needs | Motor  stereotypies, trolling | Level 3 | Moderate  (few sentences  echolalia) | Present  (IQ=52) | Hetero-aggressivity | OCD |
| Case 10 | M | 24 | Unintelligible speech, no verbal | Motor stereotypies, strolling | Level 3 | Severe  (nonverbal) | Present  (IQ=41) | Mild  hetero-aggressivity | No |
| Case 11 | M | 23 | No verbal speech | Touching people | Level 3 | Severe (nonverbal) | Present  (IQ=49) | No | No |
| Case 12 | M | 29 | Rarely interaction, no verbal speech | Page  flipping, wandering | Level 3 | Severe (nonverbal) | Present  (IQ=41) | Moderate SIB | OCD |
| Case 13 | M | 20 | No verbal speech | Strolling, body swings,  hand clapping | Level 3 | Severe  (nonvebal) | Present  (IQ=41) | Moderate SIB, mild  hetero-aggressivity | No |
| Case 14 | F | 29 | Makes unusual approaches to meet needs | Echolalia, body swings, hand clapping | Level 3 | Moderate  (few sentences  echolalia) | Present  (IQ=47) | No | Anxiety disorder |
| Case 15 | M | 19 | Makes unusual approaches to meet needs | Strolling, body swings, hand clapping, echolalia | Level 3 | Moderate  (few sentences  echolalia) | Present  (IQ=64) | No | No |

* According to DSM-5 levels of support: Level 1 (Requiring support); Level 2 (Requiring substantial support); Level 3 (Requiring very substantial support). F: female; M: male; IQ: intellectual quotient; OCD: obsessive-compulsive disorder; SIB: self-injurious behaviors.
