# Supplementary figures and images for: An individual-supported program to enhance placement in a sheltered work environment of autistic individuals mostly with intellectual disability: a prospective observational case series in an Italian community service
Source: Front Psychiatry. 2023 Nov 2;14:1225236. doi: 10.3389/fpsyt.2023.1225236 (PMC10651717; doi:10.3389/fpsyt.2023.1225236)

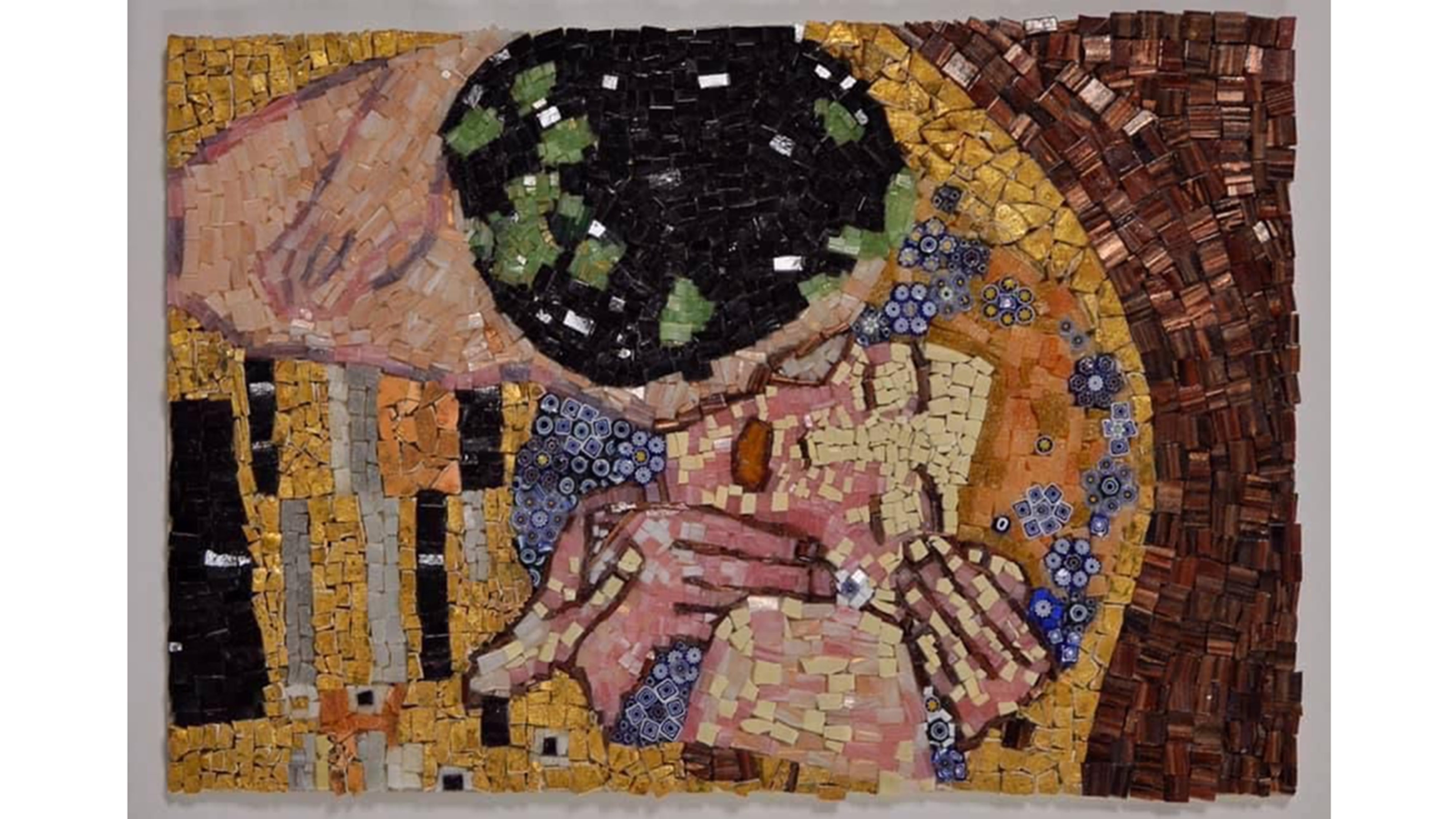

Supplement: Supplementary file 4 [file Image_1.tif]

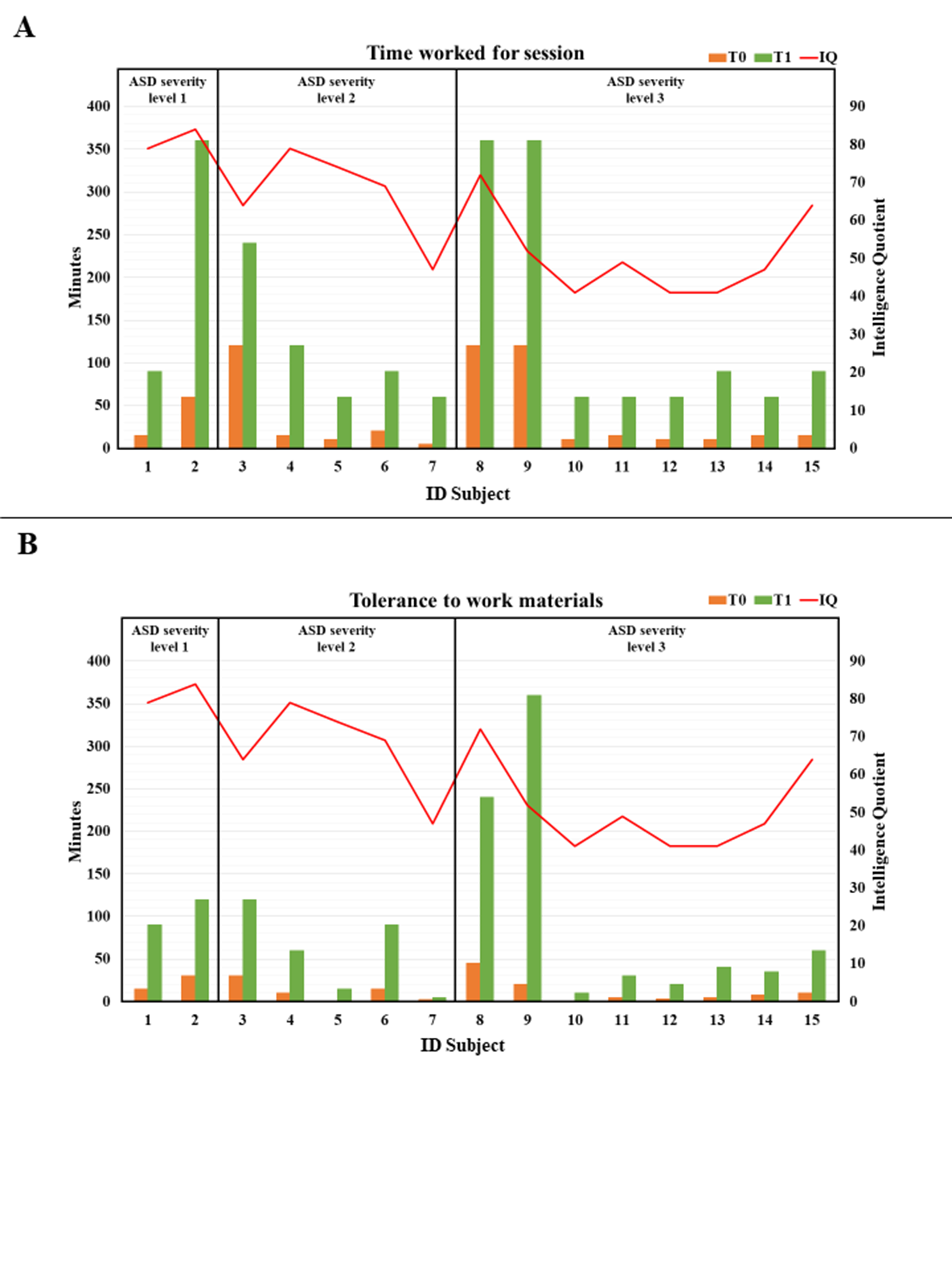

Supplement: Supplementary file 5 [file Image_2.tif]
